# The Influence of Comorbidities on Chemokine and Cytokine Profile in Obstructive Sleep Apnea Patients: Preliminary Results

**DOI:** 10.3390/jcm12030801

**Published:** 2023-01-19

**Authors:** Monika Chaszczewska-Markowska, Katarzyna Górna, Katarzyna Bogunia-Kubik, Anna Brzecka, Monika Kosacka

**Affiliations:** 1 Laboratory of Clinical Immunogenetics and Pharmacogenetics, Hirszfeld Institute of Immunology and Experimental Therapy, Polish Academy of Sciences, 50-422 Wroclaw, Poland; 2 Department of Pulmonology and Lung Oncology, Wroclaw Medical University, 53-439 Wroclaw, Poland

**Keywords:** obstructive sleep apnea, inflammatory cytokines, chemokines, hypertension, diabetes, COPD

## Abstract

Introduction: Obstructive sleep apnea (OSA) is frequently associated with a chronic inflammatory state and cardiovascular/metabolic complications. The aim of this study was to evaluate the influence of certain comorbidities on a panel of 45 chemokines and cytokines in OSA patients with special regard to their possible association with cardiovascular diseases. Material and Methods: This cross-sectional study was performed on 61 newly diagnosed OSA patients. For the measurement of the plasma concentration of chemokines and cytokines, the magnetic bead-based multiplex assay for the Luminex^®^ platform was used. Results: In the patients with concomitant COPD, there were increased levels of pro-inflammatory cytokines (CCL11, CD-40 ligand) and decreased anti-inflammatory cytokine (IL-10), while in diabetes, there were increased levels of pro-inflammatory cytokines (IL-6, TRIAL). Obesity was associated with increased levels of both pro-inflammatory (IL-13) and anti-inflammatory (IL-1RA) cytokines. Hypertension was associated with increased levels of both pro-inflammatory (CCL3) and anti-inflammatory (IL-10) cytokines. Increased daytime pCO_2_, low mean nocturnal SaO_2_, and the oxygen desaturation index were associated with increased levels of pro-inflammatory cytokines (CXCL1, PDGF-AB, TNF-α, and IL-15). Conclusions: In OSA patients with concomitant diabetes and COPD, elevated levels of certain pro-inflammatory and decreased levels of certain anti-inflammatory cytokines may favor the persistence of a chronic inflammatory state with further consequences. Nocturnal hypoxemia, frequent episodes of desaturation, and increased daytime pCO_2_ are factors contributing to the chronic inflammatory state in OSA patients.

## 1. Introduction 

Obstructive sleep apnea (OSA) is the most important sleep-breathing disorder of clinical significance. The occurrence of OSA ranges from 10% to 17% in men and from 3% to 9% in women—more frequently in older (≥50 years) persons [1]. Recurrent episodes of sleep apneas and hypopneas cause episodes of arterial oxygen desaturation and, in consequence, lead to oxidative stress, endothelial dysfunction, neurohormonal dysregulation, sleep fragmentation, and, finally, changes in the central nervous and cardiovascular systems [2,3,4,5,6,7].

Common comorbidities associated with OSA include obesity, hypertension, diabetes mellitus, and chronic obstructive pulmonary disease (COPD) [8,9]. OSA is also a risk factor of atrial fibrillation for the recurrence of atrial fibrillation after cardioversion and/or ablation [10] and for other arrhythmias [11]. OSA favors the occurrence of left heart failure [12,13] and, in some patients, pulmonary hypertension [14]. In severe cases, the risk of ischemic heart episodes [15] and stroke [16] increases. High prevalences of depression (35%) and anxiety (44%) in OSA patients have also been noted [17]. 

Obesity is typical for this disorder, being one of the causes of OSA, but—taking into account the bidirectional influences of excess weight and sleep disorders [18]—is also one of its consequences. 

There is a high risk of developing arterial hypertension in OSA patients, especially in cases of prolonged cumulative time of hypoxemia [19], independently from obesity and age [20]. OSA promotes arterial hypertension through chemoreceptor stimulation and vegetative stimulation and through the activation of the renin-angiotensin-aldosterone system [21]. The prevalence of hypertension increases with the severity of OSA [8].

Diabetes mellitus is considered the most common comorbidity of OSA, occurring in up to approximately one-third of patients [8]. There is also a bidirectional association between diabetes and OSA [22]. Coexisting diabetes mellitus increases macro- and microvascular complications of both disorders and is a risk factor for cardiovascular mortality [23].

COPD is not a clear predisposing factor for OSA, but in cases with obesity, predominant bronchitis, and fluid retention, it may constitute an increased risk for OSA [24]. The coexistence of OSA and COPD is called overlap syndrome. Coexisting COPD leads to more severe arterial oxygen desaturations during sleep and strongly worsens health-related quality of life [25]. Patients with overlap syndrome are at higher risk of developing cardiovascular complications [26]. Overlap syndrome also increases mortality [25]. 

Thus obesity, hypertension, diabetes, and COPD—all associated with the occurrence of OSA—may constitute additional risk factors for cardiac or cerebral vascular events in the course of OSA. The incidence of cardiovascular OSA complications may be influenced by the chronic inflammatory state associated with this disorder [27]. 

The immune system profoundly contributes to cardiovascular diseases [28]. The function of the immune system strongly depends on cytokines that influence humoral and cellular reactions to infections and inflammation and helps in the interplay between immune cells and organs [29]. Cytokines are small proteins of pleiotrophic, multifunctional, and hormone-like properties; there are pro-inflammatory cytokines, anti-inflammatory cytokines, and chemotactic cytokines (chemokines) [30]. They are produced by various cells, such as lymphocytes (B and T), macrophages, platelets, fibroblasts, and endothelial cells [31]. Chemokines constitute a large superfamily of ligands and receptors, participating in immunological and inflammatory disorders [32], as well as in neurobiological processes [33]. Both cytokines and chemokines strongly influence cardiovascular diseases [34,35].

No specific biomarker or group of biomarkers has been found to be associated with OSA and cardiovascular diseases. It can be hypothesized that disequilibrium in pro-inflammatory and anti-inflammatory cytokines and chemokines may be considered one of the possible pathways linking OSA and its cardiovascular complications. The aim of our study was to assess a panel of 45 chemokines and cytokines in a group of newly diagnosed OSA patients with special regard to their possible association with concomitant diseases and the severity of sleep hypoxemia.

## 2. Materials and Methods

### 2.1. Patients and Controls

In this cross-sectional study, 61 patients (F/M = 10/51) with OSA syndrome were investigated. Inclusion criteria were as follows: Age > 40 years, diagnosis based on in-hospital polysomnography (PSG), and no previous OSA treatment. Exclusion criteria encompassed a lack of agreement for participation in the study and the absence of any unstable or acute disease.

The age of the patients was 58.61 ± 11.09 years. There were 13 patients (21%) with mild OSA, 9 patients with moderate OSA (15%), and 39% (64%) with severe OSA. The mean apnea–hypopnea index (AHI) was. 42.13 ± 25.40/h and the oxygen desaturation index (ODI) was 38.92 ± 27.02/h. In all patients, daytime arterialized capillary blood gas studies were performed, and partial pressure of oxygen (pO_2_) and partial pressure of CO_2_ (pCO_2_) were analyzed.

There were 42 obese (body mass index, BMI > 30 kg/m^2^) patients (69%) and 19 non-obese patients (31%), including 16 overweight patients and 3 normal-weight patients. There were 7 patients (11%) with overlap syndrome, 16 patients (26%) with diabetes mellitus, and 43 patients (70%) with arterial hypertension. The comparison of clinical data in the groups of patients with and without comorbidities is shown in Table 1. Hypertensive patients were older than normotensive patients, diabetic patients were more obese than non-diabetic patients, overlap patients had lower mean nocturnal SaO_2_ and lower daytime pO_2_ than the patients without COPD, and obese patients had higher AHI and lower daytime pO_2_ than non-obese patients.

All participants in the study provided written informed consent. The study was approved by the local Ethics Committee (No 1082/2021), and all the procedures were in accordance with the ethical standards of the Helsinki Declaration, as revised in 2013.

### 2.2. Polysomnography 

All the patients underwent in-hospital polysomnography using the Alice 6 LDe Polysomnographic Sleep System (Philips Respironics, Monroeville, PA, USA). During 8 h of nocturnal sleep, the following parameters were measured: Airflow with the use of an oronasal thermal sensor and a nasal pressure sensor, chest and abdomen movements, oxygen saturation using a finger clip sensor for respiration, and electroencephalography, electromyography, and electrooculography for sleep stages. The following parameters were analyzed: AHI, ODI, mean arterial oxygen saturation (SaO_2_) during sleep, and minimal SaO_2_ at the end of sleep apnea/hypopnea episodes. Apneas were defined as the complete cessation of airflow for >10 s with concomitant respiratory movements of the chest and diaphragm, and hypopneas were defined as 30–50% reduction in oronasal airflow for >10 s associated with desaturation >3% or with arousal. Manual scoring was carried out after automatic scoring, according to the American Association of Sleep Medicine criteria [36]. OSA syndrome diagnosis was based on AHI >5/h and the presence of symptoms such as excessive daytime somnolence and daytime fatigue with concomitant choking and recurrent awakenings during sleep.

### 2.3. Chemokine and Cytokine Serum Levels

Serum samples were collected using BD Vacutainer SSTTM II Advance tubes (Becton Dickinson, Franklin Lakes, NJ, USA) from all participants and stored at −20 °C. Samples were then thawed and screened for the simultaneous detection of 45 chemokines and cytokines with the use of a customized Human XL Cyt Disc Premixed Mag Luminex Perf Assay Kit (R&D Systems Inc., Minneapolis, MN, USA). Analyses were performed according to the manual provided by the manufacturer. Serum samples were not diluted for the experiment. For analysis purposes, the Luminex 200 instrument (Luminex Corp., Austin, TX, USA) was used. 

The concentrations of the following proteins were measured: B7-H1 (PD-L1), CCL11 (Eotaxin), CCL19 (MIP-3-ß), CCL2 (MCP-1), CCL20 (MIP-3-α), CCL3 (MIP-1-α), CCL4 (MIP-1-ß), CCL5 (RANTES), CD40Ligand (TNFSF5), CX3CL1 (Fractalkine), CXCL1 (GRO-α), CXCL10 (IP-10), CXCL2 (GRO-ß), EGF, FGF-basic, Flt-3 Ligand, G-CSF, GM-CSF, Granzyme B, IFN-α, IFN-ß, IFN-γ, IL-1-α (IL-1F), IL-1-ß (IL-1F2), IL-1ra (IL-1F3), IL-2, IL-3, IL-4, IL-5, IL-6, IL-7, IL-8 (CXCL8), IL-10, IL-12p70, IL-13, IL-15, IL-17A, IL-17E/IL-25, IL-33, PDGF-AA, PDGF-AB (PDGF-BB), TGF-α, TNF-α, TRAIL, and VEGF.

A list of the cytokines and chemokines studied with official names and gene locus is presented in Table 2. A list of the cytokines/chemokines with their function related to inflammation and cardiovascular diseases [37,38,39,40,41,42,43,44,45,46,47,48,49,50,51,52,53,54,55,56,57,58,59,60,61,62,63,64,65,66,67,68,69,70,71,72,73,74,75,76,77,78,79,80,81] is presented in Table 3. There were no valid experimental results in the case of the following 7 cytokines/chemokines: B7-H1/PD-L1, IFN-ß, IL-3, IL-12 p70, IL-17A, IL-17E/IL-25, and IL-33, which is why these cytokines/chemokines were not analyzed in further statistical analysis.

### 2.4. Statistical Analysis

Results of the serum cytokine and chemokine level assessment were related to clinical data. All statistical analyses were performed using STATISTICA 13 software (StatSoft. Inc., Tulsa, OK, USA). The U Mann–Whitney test for two independent samples was used, and for correlation analyses, the Spearman’s Rho correlation test was used. In all calculations, the statistical significance was considered at *p* < 0.05. The effect size was measured as either the standardized mean difference (β) or Spearman’s Rho correlation coefficient (rs).

## 3. Results

The comparison of the concentrations of the chemokines and cytokines in the groups of patients with and without comorbidities is shown in Table 4. In obese patients, there were increased concentrations of IL-1ra and IL-13 compared with non-obese patients. In overlap patients, there were increased concentrations of CCL11 and CD40 ligands but decreased concentrations of IL-10 compared with patients without concomitant COPD. 

In hypertensive patients, there were increased concentrations of CCL3 and IL-10 compared with normotensive patients. In diabetic patients, there were increased concentrations of IL-6 and TRAIL compared with non-diabetic patients.

The comparison of OSA patients without any other diseases with OSA patients with only hypertension also demonstrated increased concentrations of CCL3 and, additionally, increased concentrations of CX3CL1/Fractalkine and IL-7 (Table 5).

Correlations between chemokine/cytokine levels and BMI, daytime pO_2_ and pCO_2_, and parameters indicating the severity of OSA (AHI, ODI, mean SaO_2_, and minimal SaO_2_) are shown in Table 6. There was a positive correlation between BMI and IL-1ra, and IFN-γ was negatively correlated with BMI. There was a positive correlation between daytime pO_2_ and TRAI and an inverse correlation between pCO_2_ and TRAIL. Daytime pO2 also positively correlated with the concentration of CCL11 and negatively correlated with the concentration of IL-1ra; daytime pCO_2_ was positively correlated with the concentration of CXCL1 and PDGF-AB. There was a positive correlation between ODI and TNF-α, as well as between mean SaO_2_ during sleep and IL-15, with no influences of AHI or minimal SaO_2_ at the end of sleep apneas/hypopneas.

## 4. Discussion 

There were differences in the concentrations of some chemokines/cytokines in patients with and without obesity, COPD, hypertension, and diabetes mellitus, as well as differences associated with daytime gas exchange and sleep hypoxemia.

Obesity had some influence on the cytokine/chemokine profile as shown by higher concentrations of IL-1ra and IL-13 in obese compared to non-obese OSA patients. In addition, there was a positive correlation between IL-1RA and BMI. IL-1RA has anti-inflammatory properties [82]. In the previous studies, obese, otherwise healthy persons also had elevated IL-1RA levels [72]. In patients with rheumatoid disease, IL-1ra concentrations positively correlated with BMI [67]. In the OSA patients, IL-1ra levels were also increased, and weight loss resulted in a decrease in its expression [82]. Increased levels of IL-1RA in obese OSA patients may be regarded as a protective factor. This may also confirm the negative correlation between IFN-γ and BMI. IFN-γ belongs to pro-inflammatory cytokines [83]. In adult OSA patients, elevated levels of IFNγ were found in the group with concomitant coronary heart disease [84]. In children with OSA, IFN-γ negatively correlated with cardiac function [85]. 

Overlap syndrome was associated with increased levels of the CCL11 and CD40 ligands, as well as decreased IL-10 levels. In COPD patients, there is a broad dysregulation of chemokines, including—as seen in our study in overlap patients—increased levels of CCL11 [86]. CCL11 has pro-inflammatory properties [87]. 

Increased levels of the CD40 ligand were observed in COPD patients, negatively correlating with ventilatory impairment [88]. This cytokine belongs to pro-inflammatory cytokines [89].

Decreased concentrations of Il-10 in stable COPD patients were found [90]. This is in line with the pathogenesis of COPD, as IL-10 is an anti-inflammatory cytokine [91]. In obese COPD patients, the levels of IL-10 were not decreased, indicating more severe inflammation than in non-obese COPD patients [92]. However, in some studies, COPD patients had elevated Il-10 levels [93].

In our OSA patients with concomitant arterial hypertension, there were increased concentrations of CCL3 and IL-10. In addition, the comparison of OSA patients without any other disease with OSA patients with only hypertension also demonstrated increased levels of CXCL2/GRO-ß and IL-7. In other studies, in children with primary hypertension, the serum levels of CCL3 were not different than in normotensive children [94]. Circulating levels of CCL3 (as well as CXCL10 and CD40 ligands) were associated with heart failure [50]. CCL3 has pro-inflammatory properties [95]. The role of inflammation in hypertension is still incompletely explained. There are numerous associations between changes in blood pressure and inflammatory mediators, including—as seen in our study—IL-10 and IL-7, but also interferon-γ, GM-CSF, IL-4, IL-6, IL-7, IL-8, IL-10, IL-12, IL-13, IL-17A, IL-21, IL-23, MIP-1α, and MIP-1β [96]. CXCL-2 belongs to the cytokines involved in the pathogenesis of some cardiovascular diseases as acute myocardial infarction, atherosclerosis, obesity, diabetes, and ischemic stroke [40].

Decreased levels of IL-10 were observed in OSA patients [97]. Differences in IL-10 concentrations in the OSA patients in relation to the presence of hypertension were found, with lower IL-10 concentrations in hypertensive than in normotensive patients [98]. In hypertensive patients, IL-10 was decreased [99]. IL-10 belongs to anti-inflammatory cytokines [100]. 

The influence of diabetes on the cytokine profile in our OSA patients was shown by increased levels of IL-6 and TRAIL. The data on IL-6 concentrations in OSA patients are contradictory. IL-6 was found to be increased in OSA patients compared to non-OSA patients, either obese or non-obese [101]. In another study, no significant differences in IL-6 were found in OSA and non-OSA patients [102]. In patients with OSA and diabetes, increased IL-6 levels were observed [71]. There is an influence of obesity on IL-6 levels, as its levels are positively correlated with BMI [103]. An increased IL-6 level was an independent predictor of type 2 diabetes and played an important role in inflammation, insulin resistance, and beta-cell dysfunction [104]. IL-6 belongs to pro-inflammatory cytokines [97].

TRAIL induces an inflammatory response, which stimulates the expression of chemokines and cytokines, including IL-6 [105]. Diabetic patients had decreased TRAIL levels compared to healthy controls [106,107]. There is a negative correlation between TRAIL levels and cardiovascular risk [80]. Previous studies indicated the importance of TRAIL in the development and progression of diabetes [108]. It is implicated in the regulation of T cell activation and suppresses the inflammatory process in many autoimmune diseases [109]. This modulation of the immune system also protects against diabetes [108]. TRIAL has pro-inflammatory properties [110].

In our study, in OSA patients, there was a positive correlation between TRAIL and daytime pO_2_, as well as an inverse correlation between TRAIL and daytime pCO_2_. TRAIL is a factor involved in the development of pulmonary hypertension [111,112]—a condition that may be a consequence of alveolar hypoxia and chronic hypoxemia. In OSA patients, repetitive sleep apneas and hypopneas cause alveolar hypoxia, and in cases with chronic daytime hypoxemia, pulmonary hypertension develops [113]. 

There was a positive correlation between pO_2_ and the levels of both IL-1ra and CCL11, indicating an increase in both anti-inflammatory and pro-inflammatory actions along with improved daytime gas exchange in OSA patients.

On the other hand, however, increasing daytime pCO_2_ was positively correlated with the level of CXCL1 and PDGF-AB. As both CXCL1 [114] and PDGF-AB [115] have pro-inflammatory properties, this observation indicates that the tendency to hypoventilation, as shown by increasing pCO_2_, is associated with increased inflammatory status in OSA patients.

There was a positive correlation between IL-15 and mean nocturnal SaO_2_. IL-15 belongs to pro-inflammatory cytokines [116], which indicates the association between sleep hypoxemia as a factor contributing to a chronic inflammatory state. This also confirms the positive correlation between ODI and TNF-α. TNF-α belongs to pro-inflammatory cytokines [117], which play an important role in OSA. Its serum levels increase with OSA severity and correlate with the frequency of apnea and hypopnea [118].

Our study has certain limitations. First, we divided the group of OSA patients into subgroups and compared the subgroups of OSA patients with and without comorbidities, i.e., obesity, hypertension, diabetes mellitus, and COPD, but did not compare these comorbidities with control groups. Second, the compared groups were relatively small. Moreover, the subgroups were not “pure”, e.g., all diabetic OSA patients also had hypertension, and obesity was diagnosed in 69% of our patients, thus some obese patients had to be included in other subgroups.

The main strength of the study is that in our OSA patients with the most common comorbidities, a significantly higher number of cytokines/chemokines were concomitantly measured. To the best of our knowledge, such an extensive study on cytokines and chemokines in OSA patients has not been performed.

In summary, the chemokine/cytokine profile in OSA patients with concomitant diseases indicates the inflammatory status in overlap syndrome, as shown by increased levels of pro-inflammatory proteins (CCL11, CD-40 ligand) and decreased anti-inflammatory protein (IL-10), and in diabetes, as shown by increased levels of pro-inflammatory cytokines (IL-6, TRIAL). There is an increase in the levels of both pro-inflammatory and anti-inflammatory cytokines in OSA patients with obesity (IF-γ and IL-1RA, respectively) or hypertension (CCL3 and IL-10, respectively). Increasing daytime pCO_2,_ low mean nocturnal SaO_2_, and ODI are associated with increased levels of pro-inflammatory cytokines (CXCL1, PDGF-AB, IL-15, and TNF-α, respectively). 

## 5. Conclusions

In OSA patients with concomitant diabetes and COPD, elevated levels of certain pro-inflammatory and decreased levels of certain anti-inflammatory cytokines may favor the persistence of a chronic inflammatory state with further consequences. Nocturnal hypoxemia, frequent episodes of desaturation, and increased daytime pCO_2_ are the factors contributing to the chronic inflammatory state in OSA patients.

## Figures and Tables

**Table 1:** The comparison of clinical data in the groups of OSA patients with and without comorbidities.

Clinical Data	Obesity			COPD			Hypertension			Diabetes Mellitus		
	YES *N* = 42	NO *N* = 19	*p*	YES *N* = 7	NO *N* = 54	*p*	YES *N* = 43	NO *N* = 18	*p*	YES *N* = 16	NO *N* = 45	*p*
Age [years]	57.68	60.50	0.280	58.57	58.61	0.303	**60.74**	**53.50**	**0.037**	62.13	57.36	0.087
BMI [kg/m^2^]	*NA*	*NA*	*NA*	38.99	32.98	0.100	34.27	32.25	0.317	**36.23**	**32.77**	**0.050**
AHI [n/h]	**48.84**	**24.02**	**<0.001**	53.40	40.67	0.130	42.25	41.84	0.770	38.51	43.42	0.682
Mean SaO_2_ [%]	**91.68**	**94.24**	**<0.001**	**88.80**	**93.00**	**0.004**	92.15	93.38	0.097	91.76	92.79	0.075
Minimal SaO_2_ [%]	**73.00**	**83.75**	**0.002**	71.29	77.20	0.140	76.47	76.67	0.849	77.13	76.31	0.704
pO_2_ [mmHg]	69.20	71.83	0.250	**59.60**	**71.53**	**0.008**	69.30	72.10	0.562	71.06	69.59	0.999
pCO_2_ [mmHg]	42.14	40.94	0.167	49.41	40.66	0.271	42.36	40.04	0.667	41.19	42.01	0.992

COPD—chronic obstructive pulmonary disease; BMI—body mass index; AHI—apnea hypopnea index; mean SaO_2_—mean arterial oxygen saturation during sleep; minimal SaO_2_—minimal arterial oxygen saturation at the end of sleep apneas and hypopneas; pO_2_—daytime partial pressure of oxygen; pCO_2_ daytime partial pressure of carbon dioxide; *p*—probability. Age, BMI, AHI, SaO_2_, and pO_2_ are presented as mean values across all OSA patients. Mann–Whitney U Test was used for calculations. Statistically significant results (*p* < 0.05) are shown in bold.

**Table 2:** The list of chemokines and cytokines studied.

Cytokine/Chemokine	Official Symbol	Official Name	Gene Locus
B7-H1/PD-L1	CD274	CD274 molecule	9p24.1
CCL11/eotaxin	CCL11	C-C motif chemokine ligand 11	17q12
CCL19/MIP-3-ß	CCL19	C-C motif chemokine ligand 19	9p13.3
CCL2/MCP-1	CCL2	C-C motif chemokine ligand 2	17q12
CCL20/MIP-3-α	CCL20	C-C motif chemokine ligand 20	2q36.3
CCL3/MIP-1-α	CCL3	C-C motif chemokine ligand 3	17q12
CCL4/MIP-1-ß	CCL4	C-C motif chemokine ligand 4	17q12
CCL5/RANTES	CCL5	C-C motif chemokine ligand 5	17q12
CD 40 Ligand/TNFSF5	CD40LG	CD40 ligand	Xq26.3
CX3CL1/fractalkine	CX3CL1	C-X3-C motif chemokine ligand 1	16q21
CXCL1/GRO α	CXCL1	C-X-C motif chemokine ligand 1	4q13.3
CXCL10/IP-10	CXCL10	C-X-C motif chemokine ligand 10	4q21.1
CXCL2/GRO ß	CXCL2	C-X-C motif chemokine ligand 2	4q13.3
EGF	EGF	epidermal growth factor	4q25
FGF-basic	FGF2	fibroblast growth factor 2	4q28.1
Flt-3 ligand	FLT3LG	fms related tyrosine kinase 3 ligand	19q13.33
G-CSF	CSF3	colony stimulating factor 3	17q21.1
GM-CSF	CSF2	colony stimulating factor 2	5q31.1
granzyme B	GZMB	granzyme B	14q12
IFN-ß	IFNB1	interferon beta 1	9p21.3
IL-1 α/IL-1F1	IL1A	interleukin 1 alpha	2q14.1
IL-10	IL10	interleukin 10	1q32.1
IL-12 p70	IL12A + IL12B	IL12 (p70) active heterodimer: IL-12A (p35) and IL-12B (p40)	3q25.33; 5q33.3
IL-13	IL13	interleukin 13	5q31.1
IL-15	IL15	interleukin 15	4q31.21
IL-17A	IL17A	interleukin 17A	6p12.2
IL-17E/IL-25	IL25	interleukin 25	14q11.2
IL-1-ß/IL-1F2	IL1B	interleukin 1 beta	2q14.1
IL-1ra/IL-1F3	IL1RN	interleukin 1 receptor antagonist	2q14.1
IL-2	IL2	interleukin 2	4q27
IL-3	IL3	interleukin 3	5q31.1
IL-33	IL33	interleukin 33	9p24.1
IL-4	IL4	interleukin 4	5q31.1
IL-5	IL5	interleukin 5	5q31.1
IL-6	IL6	interleukin 6	7p15.3
IL-7	IL7	interleukin 7	8q21.13
IL-8/CXCL8	CXCL8	C-X-C motif chemokine ligand 8	4q13.3
INF α	IFNA2	interferon alpha 2	9p21.3
INF γ	IFNG	interferon gamma	12q15
PDGF-AA	PDGFA	platelet derived growth factor subunit A	7p22.3
PDGF-AB/BB	PDGFB	platelet derived growth factor subunit B	22q13.1
TGF α	TGFA	transforming growth factor alpha	2p13.3
TNF-α	TNF	tumor necrosis factor	6p21.33
TRAIL	TNFSF10	TNF superfamily member 10	3q26
VEGF	VEGFA	vascular endothelial growth factor A	6p21.1

**Table 3:** The list of cytokines/chemokines with their function related to inflammation and cardiovascular diseases.

Cytokine/Chemokine	Function
B7-H1/PD-L1	Upregulated in the cells after intermittent hypoxia [37]
CCL11/eotaxin	Positively associated with vulnerable plaque burden [38]
CCL19/MIP-3-ß	Increases risk of heart failure in the patients with acute coronary syndrome [39]
CCL2/MCP-1	Involved in the pathogenesis of stroke and myocardial infarction [40]
CCL20/MIP-3-α	Biomarker of endothelial inflammation [41]
CCL3/MIP-1-α	Involved in the development of atherosclerosis [42]
CCL4/MIP-1-ß	Increased levels allow to predict cardiovascular and cerebrovascular complications of hypertension [43]; it’s inhibition may reduce endothelial inflammation [44]
CCL5/RANTES	Associated with immune cells activation in the patients with hypertension [45]
CD 40 Ligand/TNFSF5	Biomarker of carotid artery atherosclerosis [46]
CX3CL1/fractalkine	Microglial biomarker, induces bradycardic response and fall in blood pressure [47] Ruchaya 2012; mediator of chronic inflammation [48]
CXCL1/GRO α	Biomarker of carotid artery atherosclerosis [46]
CXCL10/IP-10	Associated with cardiovascular diseases, obesity [49] and heart failure [50]
CXCL2/GRO ß	Increased in cardiovascular diseases [40]
EGF	Involved in the development of pulmonary hypertension [51]
FGF-basic	Involved in the development of pulmonary hypertension [51]
Flt-3 ligand	Involved in the regulation of hematopoiesis [52]
G-CSF	Involved in cardiac repair after myocardial infarction and potential novel treatment in heart failure [53]
GM-CSF	May drive cardiovascular inflammation [54]
granzyme B	Increases in coronary artery disease [55]
IFN-ß	Anti-inflammatory cytokine [56]
IL-1 α/IL-1F1	Involved in the pathogenesis of cardiovascular diseases [57]
IL-10	Predictor of pulmonary hypertension [58]; protective effects in cardiovascular diseases in the course of diabetes [59]
IL-12 p70	Related to progression of cardiovascular diseases [60]; negative correlation with severity of coronary artery disease [61]
IL-13	Supports cardiac repair following myocardial infarction [62]
IL-15	May be protective in myocardial infarction [63]
IL-17A	Highly expressed in atherosclerotic plaques [64]
IL-17E/IL-25	Marker of severity of coronary artery disease [65]
IL-1-ß/IL-1F2	Contributes to regulation of arterial blood pressure [66]
IL-1ra/IL-1F3	Associated with increased cardiovascular risk; increases in obesity [67]
IL-2	Harmful in cardiovascular diseases in the course of diabetes [59]
IL-3	May impair cardioprotective mechanisms in the ischemia/reperfusion settings [68]
IL-33	Involved in pathophysiology of heart failure [69]
IL-4	Protective effects in cardiovascular diseases in the course of diabetes [59]; low levels in severe coronary artery disease [61]
IL-5	Facilitates heart repair after myocardial infarction [70]
IL-6	Increases in diabetes [71] and in obesity [72]
IL-7	Harmful effects in cardiovascular diseases in the course of diabetes [59]
IL-8/CXCL8	Inflammatory marker associated with mortality after myocardial infarction [73]
INF α	Pro-inflammatory cytokine [74]
INF γ	Contributes to hypertension [75]
PDGF-AA	Influence on cardiac fibroblasts function in myocardial infarction [76]
PDGF-AB/BB	Decreased levels associated with atherosclerotic plaque instability and higher risk of recurrent stroke [77]
TGF α	Involved in lung repair in COPD [78]
TNF-α	Increases in hypertension [79]
TRAIL	Negatively correlates with cardiovascular risk [80]
VEGF	Pro-angiogenetic, mitogenic and anti-apoptotic activity [81]

**Table 4:** Concentrations of chemokines/cytokines in the groups of OSA patients with and without comorbidities.

Chemokine/Cytokine [pg/mL]	Obesity			COPD			Hypertension			Diabetes Mellitus		
	YES N = 42	NO N = 19	p	YES N = 7	NO N = 54	*p*	YES N = 43	NO N = 18	*p*	YES N = 16	NO N = 45	*p*
CCL11/Eotaxin	6.74	9.86	0.121 β = −0.37	**12.66**	**7.12**	**0.044** **β = 0.66**	8.02	7.14	0.779 β = 0.10	5.90	8.42	0.624 β = −0.30
CCL19/MIP-3-ß	189.89	183.39	0.149 β = 0.04	141.65	193.73	0.453 β = −0.37	178.34	210.26	0.161 β = −0.22	168.14	194.73	0.389 β = −0.18
CCL2/MCP-1	757.41	791.98	0.834 β = −0.09	654.90	783.50	0.424 β = −0.34	716.89	892.63	0.105 β = −0.47	816.91	751.62	0.667 β = 0.17
CCL20/MIP-3-α	20.27	31.05	0.167 β = −0.26	8.21	25.83	0.149 β = −0.44	25.46	19.85	0.952 β = 0.14	27.10	22.63	0.896 β = 0.11
CCL3/MIP-1-α	28.95	45.08	0.215 β = −0.31	26.22	35.27	0.674 β = −0.17	**40.63**	**18.96**	**0.008** **β = 0.42**	41.13	31.78	0.294 β = 0.18
CCL4/MIP-1-ß	0.29	0.28	0.904 β = 0.11	0.29	0.28	0.865 β = 0.12	0.29	0.27	0.920 β = 0.25	0.32	0.27	0.267 β = 0.62
CCL5/RANTES	241.99	169.75	0.204 β = 0.39	234.30	216.23	0.952 β = 0.09	221.14	211.54	0.968 β = 0.05	245.26	208.72	0.952 β = 0.20
CD40 Ligand/TNFSF5	6.69	7.11	0.327 β = −0.10	**8.83**	**6.57**	**0.016** **β = 0.54**	7.12	6.13	0.379 β = 0.24	7.24	6.68	0.164 β = 0.13
CX3CL1/Fractalkine	0.04	0.04	0.190 β = 0	0.04	0.04	0.704 β = 0	0.04	0.05	0.787 β = −0.20	0.03	0.04	0.944 β = −0.20
CXCL1/GRO-α	0.05	0.03	0.542 β = 0.40	0.04	0.04	0.976 β = 0	0.04	0.04	0.968 β = 0	0.04	0.04	0.764 β = 0
CXCL10/IP-10	55.35	63.12	0.960 β = −0.18	54.31	58.36	0.849 β = −0.09	53.13	69.29	0.373 β = −0.38	67.95	54.32	0.234 β = 0.32
CXCL2/GRO-ß	265.03	209.31	0.802 β = 0.20	256.31	245.52	0.772 β = 0.04	259.95	215.25	0.696 β = 0.15	396.15	193.65	0.128 β = 0.72
EGF	240.86	270.86	0.280 β = −0.29	252.12	250.51	0.992 β = 0.01	250.90	250.21	0.674 β = 0.01	247.59	251.80	0.726 β = −0.03
FGF-basic	16.69	16.01	0.881 β = 0.02	10.27	17.27	0.704 β = −0.21	16.41	16.60	0.834 β = −0.01	12.35	17.93	0.749 β = −0.17
Flt-3 Ligand	0.11	0.10	0.363 β = 0.25	0.10	0.11	0.881 β = −0.25	0.11	0.11	0.667 β = 0	0.10	0.11	0.818 β = −0.25
G-CSF	7.32	7.06	0.960 β = 0.07	7.07	7.26	0.711 β = −0.05	7.27	7.15	0.682 β = 0.03	7.45	7.16	0.645 β = 0.08
GM-CSF	0.002	0.005	0.726 β = −0.75	0.002	0.003	0.756 β = −0.25	0.002	0.004	0.952 β = −0.50	0.002	0.003	0.667 β = −0.25
Granzyme B	2.86	9.35	0.234 β = −0.49	3.02	5.24	0.726 β = −0.17	5.69	3.31	0.779 β = 0.18	1.28	6.30	0.810 β = −0.38
IFN-α	1.03	1.01	0.379 β = 0.02	0.90	1.04	0.711 β = −0.14	0.93	1.24	0.555 β = −0.31	0.98	1.04	0.660 β = −0.06
IFN-γ	0.31	7.79	0.779 β = −0.58	0.16	3.10	0.535 β = −0.24	1.81	5.04	0.741 β = −0.26	0.35	3.62	0.928 β = −0.27
IL-1-α/IL-1F1	0.61	0.24	0.516 β = 0.29	0.27	0.51	0.889 β = −0.20	0.58	0.25	0.610 β = 0.27	0.60	0.44	0.238 β = 0.13
IL-1-ß/IL-1F2	0.33	0.28	0.603 β = 0.12	0.55	0.28	0.303 β = 0.67	0.38	0.15	0.197 β = 0.57	0.33	0.30	0.631 β = 0.07
IL-1ra/IL-1F3	**589.99**	**380.29**	**0.007** β = 0.57	497.34	524.34	0.674 β = −0.07	541.60	472.59	0.548 β = 0.18	628.38	483.14	0.509 β = 0.39
IL-2	1.07	1.26	0.610 β = −0.13	1.30	1.11	0.603 β = 0.13	1.03	1.38	0.569 β = −0.25	0.90	1.21	0.936 β = −0.22
IL-4	0.09	0.10	0.267 β = −0.05	0.04	0.10	0.873 β = −0.30	0.08	0.11	0.952 β = −0.15	0.07	0.10	0.516 β = −0.15
IL-6	3.13	1.97	0.603 β = 0.36	2.97	2.72	0.603 β = 0.08	2.87	2.46	0.535 β = 0.13	**4.02**	**2.29**	**0.009** **β = 0.55**
IL-7	5.74	4.53	0.180 β = 0.39	5.00	5.39	0.478 β = −0.12	5.05	6.05	0.180 β = −0,32	4.55	5.63	0.128 β = −0.36
IL-8/CXCL8	46.07	58.39	0.936 β = −0.13	52.93	49.75	0.653 β = 0.03	53.94	40.98	0.726 β = 0.14	46.90	51.25	0.810 β = −0.04
IL-10	0.12	0.01	0.795 β = 0.21	**0.004**	**0.09**	**0.024** **β = −0.17**	**0.113**	**0.019**	**0.036** **β = 0.18**	0.267	0.020	0.889 β = 0.49
IL-13	**0.02**	**0.01**	**0.043** **β = 1.00**	0.02	0.02	0.478 β = 0	0.01	0.02	0.289 β = 0.66	0.02	0.02	0.575 β = 0
IL-15	0.15	0.18	0.327 β = −0.10	0.22	0.16	0.542 β = 0.20	0.14	0.22	0.849 β = −0.26	0.15	0.17	0.496 β = −0.06
PDGF-AA	9144.1	11,312.6	0.131 β = −0.44	9753.3	9868.2	0.992 β = −0.02	9473.9	10,765.5	0.298 β = −0.26	8995.5	10,160.7	0.529 β = −0.23
PDGF-AB/BB	9366.5	19,478.7	0.704 β = −0.35	38781.2	9298.8	0.928 β = 1.02	14,395.4	8589.0	0.756 β = 0,20	9290.1	13,888.0	0.912 β = −0.16
TGF-α	2.27	2.79	0.610 β = −0.17	1.66	2.54	0.944 β = −0.23	2.44	2.46	0.889 β = −0.01	2.44	2.44	0.873 β = 0
TNF-α	1.88	1.72	0.912 β = 0.05	2.18	1.78	0.936 β = 0.14	1.79	1.92	0.756 β = −0.04	2.23	1.68	0.764 β = 0.19
TRAIL	0.02	0.02	0.667 β = 0	0.01	0.02	0.226 β = −0.55	0.02	0.02	0.516 β = 0	**0.03**	**0.01**	**0.007** **β = 2**
VEGF	145.73	112.69	0.928 β = 0.16	117.90	137.10	0.810 β = −0.10	139.15	124.72	0.719 β = 0.07	113.36	142.55	0.667 β = −0.14

COPD—Chronic Obstructive Pulmonary Disease. The values are presented as mean across all OSA patients. Mann–Whitney U Test was used for calculations. β—standardized mean difference. Statistically significant results (*p* < 0.05) are shown in bold.

**Table 5:** Associations between cytokine levels in OSA patients without any other diseases vs. in OSA patients with concomitant only disorders: Obesity defined by BMI > 30 kg/m^2^ or only with hypertension. Levels of cytokines in patients with/without given disorder are presented as mean values. Mann–Whitney U Test was used for calculations. β—standardized mean difference. Statistically significant results (*p* ≤ 0.05) are shown in bold.

Cytokine	1	2	3		
	OSA without Comorbidities	OSA + Obesity	OSA + Hypertension	1 vs. 2	1 vs. 3
	N = 7	N = 11	N = 9	*p*	*p*
CCL11/Eotaxin	12.63	3.65	6.19	0.779 β = 1.07	0.064 β = 0.75
CCL19/MIP-3-beta	175.40	232.44	213.74	**0.015** **β** **= −0.34**	0.596 β = −0.33
CCL2/MCP-1	814.74	942.19	618.90	**0.023** **β** **= −0.40**	0.342 β = 0.62
CCL20/MIP-3-alpha	30.99	12.76	38.90	0.107 β = 0.56	0.674 β = −0.17
CCL3/MIP-1-alpha	13.32	22.54	71.76	0.063 β = −0.34	**0.030** **β** **= −0.76**
CCL4/MIP-1-beta	0.28	0.27	0.28	0.976 β = 0.20	0.833 β = 0
CCL5/RANTES	146.60	252.86	152.13	**0.012** **β** **= −0.59**	0.912 β = −0.08
CD40 Ligand/TNFSF5	6.95	5.60	6.61	0.147 β = 0.42	0.913 β = 0.1
CX3CL1/Fractalkine	0.02	0.07	0.06	0.976 β = −0.71	**0.044** **β** **= −0.88**
CXCL1/GRO-alpha	0.02	0.05	0.03	0.976 β = −0.75	0.749 β = −0.5
CXCL10/IP-10	79.36	62.88	43.96	**0.056** **β** **= 0.28**	0.342 β = 0.68
CXCL2/GRO-beta	139.60	263.39	154.86	**0.007** **β** **= −0.62**	0.674 β = −0.15
EGF	260.27	243.81	281.53	**0.044** **β** **= 0.16**	0.748 β = −0.25
FGF-basic	26.54	10.27	10.36	0.976 β = 0.62	0.912 β = 0.57
Flt-3 Ligand	0.11	0.11	0.11	0.976 β = 0	0.834 β = 0
G-CSF	8.13	6.53	5.99	0.298 β = 0.41	0.342 β = 0.64
GM-CSF	0.02	0.02	0.02	0.976 β = 0	0.459 β = 0
Granzyme B	6.69	1.16	12.95	0.322 β = 0.58	0.562 β = −0.32
IFN-alpha	1.06	1.36	1.12	0.107 β = −0.18	0.091 β = −0.05
IFN-gamma	12.22	0.42	7.68	0.779 β = 0.59	0.912 β = 0.18
IL-1-alpha/IL-1F1	0.18	0.31	0.26	0.976 β = −0.59	0.749 β = −0.47
IL-1-beta/IL-1F2	0.12	0.15	0.38	0.987 β = −0.21	0.596 β = −0.59
IL-1ra/IL-1F3	386.32	527.49	356.16	**0.005** **β** **= −0.54**	0.834 β = 0.16
IL-2	1.09	1.55	1.14	0.230 β = −0.24	0.873 β = −0.03
IL-3	0.02	0.001	0.01	0.989 β = 0.95	0.912 β = 0.33
IL-4	0.02	0.17	0.20	0.987 β = −0.71	0.749 β = −0.60
IL-6	2.29	2.56	1.71	0.271 β = −0.15	0.222 β = 0.48
IL-7	3.36	7.76	5.72	**<0.001** **β** **= −1.41**	**0.044** **β** **= −0.94**
IL-8/CXCL8	24.24	51.63	89.63	**0.026** **β** **= −0.40**	0.167 β = −0.55
IL-10	0.01	0.02	0.01	0.976 β = −0.50	0.167 β = 0
IL-12 p70	0.001	0.002	0.001	0.976 β = 1	0.748 β = 0
IL-13	0.02	0.02	0.01	0.987 β = 0	0.395 β = 0.5
IL-15	0.47	0.04	0.01	0.978 β = 1.07	0.167 β = 1.06
IL-33	0.003	0.001	0.002	0.976 β = 0.66	0.912 β = 0.33
PDGF-AA	10,438.84	10,973.37	12,565.74	0.070 β = −0.10	0.674 β = −0.40
PDGF-AB/BB	10,774.76	7197.99	7242.01	0.342 β = 0.62	0.395 β = 0.57
TGF-alpha	3.59	1.74	3.20	0.211 β = 0.46	0.711 β = 0.08
TNF-alpha	1.67	2.07	1.83	0.177 β = −0.16	1.000 β = −0.05
TRAIL	0.02	0.01	0.01	0.976 β = 0.76	0.749 β = 0.76
VEGF	80.92	152.58	122.17	**0.003** **β** **= −0.78**	0.204 β = −0.56

**Table 6:** Correlations of chemokine/cytokine levels with clinical data.

Chemokine/Cytokine	BMI	pO_2_	pCO_2_	AHI	Mean SaO_2_	Minimal SaO_2_	ODI
CCL11/Eotaxin	*p* = 0.553 r_s_ = −0.103	***p*** ** = 0.019** **r_s_ = 0.419**	*p* = 0.331 r_s_ = −0.180	*p* = 0.103 r_s_ = −0.280	*p* = 0.714 r_s_ = 0.064	*p* = 0.636 r_s_ = 0.062	*p* = 0.350 r_s_ = −0.162
CCL19/MIP-3-ß	*p* = 0.054 r_s_ = 0.247	*p* = 0.876 r_s_ = −0.021	*p* = 0.978 r_s_ = 0.003	*p* = 0.346 r_s_ = 0.123	*p* = 0.428 r_s_ = 0.103	*p* = 0.612 r_s_ = 0.066	*p* = 0.340 r_s_ = 0.124
CCL2/MCP-1	*p* = 0.745 r_s_ = −0.042	*p* = 0.066 r_s_ = 0.249	*p* = 0.089 r_s_ = −0.230	*p* = 0.470 r_s_ = 0.094	*p* = 0.697 r_s_ = −0.050	*p* = 0.524 r_s_ = 0.083	*p* = 0.242 r_s_ = 0.152
CCL20/MIP-3-α	*p* = 0.167 r_s_ = −0.182	*p* = 0.950 r_s_ = 0.008	*p* = 0.098 r_s_ = −0.229	*p* = 0.726 r_s_ = −0.046	*p* = 0.122 r_s_ = 0.203	*p* = 0.143 r_s_ = 0.189	*p* = 0.483 r_s_ = −0.092
CCL3/MIP-1-α	*p* = 0.333 r_s_ = −0.144	*p* = 0.711 r_s_ = 0.057	*p* = 0.641 r_s_ = −0.072	*p* = 0.795 r_s_ = −0.039	*p* = 0.310 r_s_ = −0.151	*p* = 0.969 r_s_ = 0.005	*p* = 0.921 r_s_ = 0.014
CCL4/MIP-1-ß	*p* = 0.788 r_s_ = 0.034	*p* = 0.786 r_s_ = −0.037	*p* = 0.462 r_s_ = −0.101	*p* = 0.466 r_s_ = −0.095	*p* = 0.719 r_s_ = −0.046	*p* = 0.991 r_s_ = −0.001	*p* = 0.789 r_s_ = −0.034
CCL5/RANTES	*p* = 0.548 r_s_ = 0.083	*p* = 0.487 r_s_ = 0.100	*p* = 0.650 r_s_ = −0.109	*p* = 0.930 r_s_ = 0.012	*p* = 0.910 r_s_ = −0.015	*p* = 0.842 r_s_ = 0.026	*p* = 0.952 r_s_ = −0.008
CD40 Ligand/TNFSF5	*p* = 0.885 r_s_ = −0.018	*p* = 0.352 r_s_ = −0.127	*p* = 0.383 r_s_ = −0.119	*p* = 0.664 r_s_ = −0.057	*p* = 0.697 r_s_ = 0.050	*p* = 0.522 r_s_ = 0.083	*p* = 0.447 r_s_ = −0.099
CX3CL1/Fractalkine	*p* = 0.294 r_s_ = 0.165	*p* = 0.082 r_s_ = −0.285	*p* = 0.808 r_s_ = 0.040	*p* = 0.507 r_s_ = 0.105	*p* = 0.497 r_s_ = −0.107	*p* = 0.985 r_s_ = 0.002	*p* = 0.738 r_s_ = 0.053
CXCL1/GRO-α	*p* = 0.259 r_s_ = 0.393	*p* = 0.149 r_s_ = −0.490	***p*** ** = 0.048** **r_s_ = 0.636**	*p* = 0.579 r_s_ = 0.200	*p* = 0.683 r_s_ = −0.147	*p* = 0.078 r_s_ = −0.227	*p* = 0.579 r_s_ = −0.200
CXCL10/IP-10	*p* = 0.471 r_s_ = 0.093	*p* = 0.164 r_s_ = −0.190	*p* = 0.808 r_s_ = 0.033	*p* = 0.320 r_s_ = 0.129	*p* = 0.471 r_s_ = −0.093	*p* = 0.586 r_s_ = −0.071	*p* = 0.334 r_s_ = 0.125
CXCL2/GRO-ß	*p* = 0.908 r_s_ = 0.015	*p* = 0.981 r_s_ = 0.003	*p* = 0.709 r_s_ = 0.051	*p* = 0.661 r_s_ = 0.057	*p* = 0.351 r_s_ = −0.121	*p* = 0.628 r_s_ = −0.063	*p* = 0.567 r_s_ = 0.074
EGF	*p* = 0.810 r_s_ = 0.031	*p* = 0.608 r_s_ = −0.070	*p* = 0.160 r_s_ = −0.191	*p* = 0.508 r_s_ = −0.086	*p* = 0.229 r_s_ = 0.156	*p* = 0.657 r_s_ = 0.058	*p* = 0.479 r_s_ = −0.092
FGF-basic	*p* = 0.284 r_s_ = 0.600	*p* = 0.800 r_s_ = −0.200	*p* = 0.051 r_s_ = 0.948	*p* = 0.747 r_s_ = 0.200	*p* = 0.218 r_s_ = 0.666	*p* = 0.722 r_s_ = −0.046	*p* = 0.747 r_s_ = 0.200
Flt-3 Ligand	*p* = 0.455 r_s_ = 0.097	*p* = 0.354 r_s_ = −0.127	*p* = 0.457 r_s_ = −0.102	*p* = 0.454 r_s_ = 0.097	*p* = 0.800 r_s_ = −0.032	*p* = 0.284 r_s_ = −0.139	*p* = 0.329 r_s_ = 0.126
G-CSF	*p* = 0.604 r_s_ = 0.067	*p* = 0.421 r_s_ = −0.110	*p* = 0.930 r_s_ = 0.011	*p* = 0.583 r_s_ = −0.071	*p* = 0.190 r_s_ = 0.169	*p* = 0.050 r_s_ = 0.252	*p* = 0.136 r_s_ = −0.192
GM-CSF	*p* = 0.262 r_s_ = −0.737	*p* = 1.000 r_s_ = 0.000	*p* = 0.666 r_s_ = 0.500	*p* = 0.051 r_s_ = −0.949	-	*p* = 0.826 r_s_ = 0.029	*p* = 0.262 r_s_ = −0.737
Granzyme B	*p* = 0.104 r_s_ = −0.355	*p* = 0.550 r_s_ = −0.134	*p* = 0.582 r_s_ = −0.123	*p* = 0.261 r_s_ = −0.250	*p* = 0.144 r_s_ = 0.321	*p* = 0.314 r_s_ = 0.131	*p* = 0.300 r_s_ = −0.231
IFN-α	*p* = 0.355 r_s_ = 0.120	*p* = 0.731 r_s_ = −0.041	*p* = 0.385 r_s_ = 0.119	*p* = 0.639 r_s_ = 0.051	*p* = 0.502 r_s_ = −0.087	*p* = 0.135 r_s_ = −0.192	*p* = 0.578 r_s_ = 0.072
IFN-γ	***p*** ** = 0.025** **r_s_ = −0.771**	*p* = 0.380 r_s_ = −0.441	*p* = 0.505 r_s_ = −0.343	*p* = 0.113 r_s_ = −0.602	*p* = 0.863 r_s_ = 0.073	*p* = 0.891 r_s_ = −0.018	*p* = 0.153 r_s_ = −0.554
IL-1-α/IL-1F1	*p* = 0.086 r_s_ = 0.0568	*p* = 0.943 r_s_ = −0.027	*p* = 0.887 r_s_ = 0.055	*p* = 0.748 r_s_ = −0.116	*p* = 0.237 r_s_ = 0.411	*p* = 0.955 r_s_ = 0.007	*p* = 0.529 r_s_ = −0.226
IL-1-ß/IL-1F2	*p* = 0.567 r_s_ = 0.108	*p* = 0.453 r_s_ = −0.134	*p* = 0.840 r_s_ = 0.039	*p* = 0.319 r_s_ = 0.188	*p* = 0.486 r_s_ = −0.132	*p* = 0.186 r_s_ = −0.172	*p* = 0.615 r_s_ = 0.095
IL-1ra/IL-1F3	***p*** ** = 0.002** **r_s_ = 0.396**	***p*** ** = 0.035** **r_s_ = −0.285**	*p* = 0.130 r_s_ = 0.206	*p* = 0.078 r_s_ = 0.227	*p* = 0.548 r_s_ = −0.078	*p* = 0.483 r_s_ = −0.091	*p* = 0.077 r_s_ = 0.227
IL-2	*p* = 0.811 r_s_ = 0.033	*p* = 0.291 r_s_ = 0.153	*p* = 0.812 r_s_ = 0.034	*p* = 0.827 r_s_ = 0.030	*p* = 0.864 r_s_ = −0.023	*p* = 0.225 r_s_ = −0.158	*p* = 0.726 r_s_ = 0.048
IL-3	*p* = 0.600 r_s_ = −0.400	- -	*p* = 1.000 r_s_ = 0.000	*p* = 0.200 r_s_ = −0.800	*p* = 0.200 r_s_ = 0.800	- -	*p* = 0.600 r_s_ = −0.400
IL-4	*p* = 0.178 r_s_ = −0.342	*p* = 0.217 r_s_ = 0.326	*p* = 0.883 r_s_ = 0.038	*p* = 0.138 r_s_ = 0.374	*p* = 0.355 r_s_ = −0.239	*p* = 0.392 r_s_ = −0.111	*p* = 0.241 r_s_ = 0.300
IL-6	*p* = 0.320 r_s_ = 0.135	*p* = 0.056 r_s_ = −0.265	*p* = 0.796 r_s_ = −0.036	*p* = 0.723 r_s_ = −0.048	*p* = 0.233 r_s_ = −0.161	*p* = 0.439 r_s_ = −0.101	*p* = 0.629 r_s_ = 0.065
IL-7	*p* = 0.069 r_s_ = 0.236	*p* = 0.946 r_s_ = 0.009	*p* = 0.672 r_s_ = −0.058	*p* = 0.113 r_s_ = 0.206	*p* = 0.368 r_s_ = −0.118	*p* = 0.184 r_s_ = −0.172	*p* = 0.057 r_s_ = 0.246
IL-8/CXCL8	*p* = 0.468 r_s_ = −0.097	*p* = 0.767 r_s_ = 0.042	*p* = 0.722 r_s_ = −0.050	*p* = 0.513 r_s_ = 0.088	*p* = 0.498 r_s_ = −0.091	*p* = 0.738 r_s_ = −0.044	*p* = 0.416 r_s_ = 0.109
IL-10	*p* = 0.721 r_s_ = −0.049	*p* = 0.608 r_s_ = 0.074	*p* = 0.816 r_s_ = −0.033	*p* = 0.20 r_s_ = 0.174	*p* = 0.362 r_s_ = 0.125	*p* = 0.749 r_s_ = 0.042	*p* = 0.276 r_s_ = 0.149
IL-13	*p* = 0.786 r_s_ = 0.040	*p* = 0.750 r_s_ = 0.049	*p* = 0.239 r_s_ = −0.181	*p* = 0.339 r_s_ = −0.144	*p* = 0.124 r_s_ = 0.229	*p* = 0.309 r_s_ = −0.132	*p* = 0.402 r_s_ = −0.126
IL-15	*p* = 0.702 r_s_ = −0.090	*p* = 0.498 r_s_ = −0.170	*p* = 0.691 r_s_ = −0.100	*p* = 0.938 r_s_ = −0.018	***p*** ** = 0.045** **r_s =_ 0.452**	*p* = 0.982 r_s_ = −0.003	*p* = 0.705 r_s_ = −0.090
IL-33	- -	*p* = 0.666 r_s_ = −0.500	*p* = 0.666 r_s_ = −0.500	*p* = 0.666 r_s_ = −0.500	*p* = 0.666 r_s_ = 0.500	- -	*p* = 0.666 r_s_ = −0.500
PDGF-AA	*p* = 0.600 r_s_ = −0.068	*p* = 0.902 r_s_ = 0.016	*p* = 0.738 r_s_ = 0.046	*p* = 0.183 r_s_ = −0.172	*p* = 0.117 r_s_ = 0.202	*p* = 0.691 r_s_ = 0.052	*p* = 0.120 r_s_ = −0.200
PDGF-AB/BB	*p* = 0.934 r_s_ = −0.011	*p* = 0.055 r_s_ = −0.290	***p*** ** = 0.007** **r_s_ = 0.398**	*p* = 0.542 r_s_ = 0.089	*p* = 0.235 r_s_ = −0.172	*p* = 0.577 r_s_ = −0.073	*p* = 0.626 r_s_ = 0.071
TGF-α	*p* = 0.243 r_s_ = 0.215	*p* = 0.504 r_s_ = −0.131	*p* = 0.844 r_s_ = 0.038	*p* = 0.653 r_s_ = 0.083	*p* = 0.581 r_s_ = −0.102	*p* = 0.199 r_s_ = −0.166	*p* = 0.496 r_s_ = 0.126
TNF-α	*p* = 0.377 r_s_ = 0.170	*p* = 0.692 r_s_ = 0.083	*p* = 0.149 r_s_ = 0.296	*p* = 0.110 r_s_ = 0.303	*p* = 0.387 r_s_ = −0.166	*p* = 0.462 r_s_ = 0.096	***p*** ** = 0.023** **r_s =_ 0.422**
TRAIL	*p* = 0.828 r_s_ = −0.031	***p*** ** = 0.002** **r_s_ = 0.438**	***p*** ** = 0.040** **r_s_ = −0.303**	*p* = 0.192 r_s_ = −0.187	*p* = 0.539 r_s_ = 0.088	*p* = 0.285 r_s_ = 0.139	*p* = 0.343 r_s_ = −0.136
VEGF	*p* = 0.548 r_s_ = 0.078	*p* = 0.789 r_s_ = −0.036	*p* = 0.695 r_s_ = 0.054	*p* = 0.587 r_s_ = 0.070	*p* = 0.889 r_s_ = 0.018	*p* = 0.659 r_s_ = −0.057	*p* = 0.429 r_s_ = 0.103

Spearman’s Rho correlation test was used for calculations. Statistically significant results (*p* < 0.05) are shown in bold, r_s_—Spearman’s Rho correlation coefficient, BMI—body mass index, AHI—apnea–hypopnea index, pO_2_—partial pressure of oxygen, pCO_2_—partial pressure of carbon dioxide, Mean SaO_2_—mean saturation during sleep, Minimal SaO_2_—minimal saturation at the end of apneas/hypopneas, ODI—oxygen desaturation index.

## Data Availability

The data presented in this study are available on request from the corresponding author. The data are not publicly available due to privacy.
